# Lack of evidence for *KRAS *oncogenic mutations in triple-negative breast cancer

**DOI:** 10.1186/1471-2407-10-136

**Published:** 2010-04-13

**Authors:** Alfonso Sánchez-Muñoz, Elena Gallego, Vanessa de Luque, Luís G Pérez-Rivas, Luís Vicioso, Nuria Ribelles, José Lozano, Emilio Alba

**Affiliations:** 1 Servicio de Oncología Médica, Hospital Universitario Virgen de la Victoria, Campus de Teatinos s/n, 29010 Málaga, Spain; 2 Servicio de Anatomía Patológica, Hospital Universitario Virgen de la Victoria, Campus de Teatinos s/n, 29010 Málaga, Spain; 3 Laboratorio de Investigación Biomédica (LIB-IMABIS), Hospital Universitario Virgen de la Victoria, Campus de Teatinos s/n, 29010 Málaga, Spain; 4 Dpto. de Biología Molecular y Bioquímica, Facultad de Ciencias, Universidad de Málaga, Campus de Teatinos s/n, 29071 Málaga, Spain

## Abstract

**Background:**

Mutational analysis of the *KRAS *gene has recently been established as a complementary *in vitro *diagnostic tool for the identification of patients with colorectal cancer who will not benefit from anti-epidermal growth factor receptor (EGFR) therapies. Assessment of the mutation status of *KRAS *might also be of potential relevance in other EGFR-overexpressing tumors, such as those occurring in breast cancer. Although *KRAS *is mutated in only a minor fraction of breast tumors (5%), about 60% of the basal-like subtype express EGFR and, therefore could be targeted by EGFR inhibitors. We aimed to study the mutation frequency of *KRAS *in that subtype of breast tumors to provide a molecular basis for the evaluation of anti-EGFR therapies.

**Methods:**

Total, genomic DNA was obtained from a group of 35 formalin-fixed paraffin-embedded, triple-negative breast tumor samples. Among these, 77.1% (27/35) were defined as basal-like by immunostaining specific for the established surrogate markers cytokeratin (CK) 5/6 and/or EGFR. *KRAS *mutational status was determined in the purified DNA samples by Real Time (RT)-PCR using primers specific for the detection of wild-type *KRAS *or the following seven oncogenic somatic mutations: Gly12Ala, Gly12Asp, Gly12Arg, Gly12Cys, Gly12Ser, Gly12Val and Gly13Asp.

**Results:**

We found no evidence of *KRAS *oncogenic mutations in all analyzed tumors.

**Conclusions:**

This study indicates that *KRAS *mutations are very infrequent in triple-negative breast tumors and that EGFR inhibitors may be of potential benefit in the treatment of basal-like breast tumors, which overexpress EGFR in about 60% of all cases.

## Background

Breast cancer is a complex and heterogeneous disease that includes tumors of variable prognosis and clinical response to treatments [1]. Standard breast tumor classification has long relied on morphological and anatomical criteria such as tumor size and extension (TNM staging), histopathological features (tumor grade) and expression of protein markers such as the estrogen receptor (ER), progesterone receptor (PR) and the human epidermal growth factor receptor 2 (HER2) oncogene [1,2]. While these parameters may correlate well with survival in some patients, their value as prognostic and predictive factors is limited given the fact that patients with similar tumors often have a different clinical progression and treatment response [1,2]. The existence of such differences in the clinical outcome of breast cancer patients can be explained by intrinsic tumor variability at the molecular level [3-7]. In two landmark studies, Perou *et al*. and Sorlie *et al*. [3,5] identified five distinct "intrinsic" subtypes of breast cancer by hierarchical cluster analysis of microarray gene expression data: luminal A and luminal B [both estrogen receptor-positive (ER+)], HER2 overexpressing (HER2+), normal breast-like and basal-like. These subtypes are associated with different clinical outcomes, with the HER2+ and basal-like subtypes being more agressive and having poor prognoses [5,8]. The term triple-negative is frequently used as synonymous for basal-like, since these tumors lack expression of ER, PR and HER2 [9,10]. However, not all triple-negative tumors are basal-like while most basal-like tumors are triple-negative [4,10]. Triple-negative tumors are found in only 15% of all breast cancer patients and the incidence varies by race and age. In particular, the basal subtype represents 10-14% of all breast cancers in Caucasian women and 20-37% in African American patients [11,12]. Despite their low incidence, triple-negative breast cancer represent a major clinical challenge due to the high mortality associated with the disease [4].

At the immunohistochemical level, the basal-like subtype express a group of proteins similar to those expressed in the basal -hence the name- ephithelial cells of the mammary gland. These include cytokeratins (CK) 5, 6 and 17, epidermal growth factor receptor (EGFR), caveolin, Ki-67, c-KIT and αβ-crystalin [3,5,13,14]. Most of basal-like tumors are highly proliferative, show high histologic grade and are associated with a higher incidence of mutations in *BRCA1 *and *TP53 *[15]. Clinically, these tumors are agressive and tend to form metastasis in the lungs or in the brain [16,17]. Similar to the HER2+, the triple-negative subtype shows responsiveness to chemotherapy with taxanes and anthracyclines [18,19]. It is typically associated with a bad prognostic, as defined by reduced disease-free survival (DFS) and overall survival (OS) rates [16,17]. Hormone therapies and anti-HER2 therapies are innefective in the treatment of triple-negative breast cancer and thus, searching for new drug targets selective for this subtype of tumors is a major challenge in modern oncology.

Near 60% of basal-like tumors express the epidermal growth factor receptor (EGFR) and, therefore are potential targets of EGFR inhibitors such as the monoclonal antibodies cetuximab and panitumumab or the small molecule inhibitors gefitinib and erlotinib. [20,21]. Signals initiated at the EGFR are transmited intracellularly by members of the GTPase Ras family of proteins, which function as molecular switches in the transduction of proliferative and differentiating signals [22]. Members of this family include *KRAS*, *HRAS*, *NRAS *and *RRAS*, with *KRAS *being a mammalian homolog of the Kirsten ras oncogene [23]. Ras proteins are activated by guanine nucleotide exchange factors (GEFs), allowing release of bound GDP and binding of cytosolic GTP. Once activated, they function at the plasma membrane by recruiting several signaling proteins such as RAF, PI 3-kinase and RalGDS [24]. The low intrinsinc GTPase activity of Ras is increased by interaction with GAP (GTPase activating protein) which hydrolizes bound GTP and turns off Ras signaling [24]. Several oncogenic mutations have been described in the *KRAS *gene wich result in its constitutive activation and in autonomous, non-regulated proliferation of the transformed cells as well as their resistance to apoptosis [22]. Somatic *KRAS *mutations are found in pancreatic cancer (60% of tumors), colon cancer (32%), lung cancer (17%) and, with a much lower incidence (5%), in leukemias and breast cancer [24]. Germline mutations in *KRAS *are associated with Noonan syndrome and cardio-facio-cutaneous syndrome [25,26]. Anti-EGFR therapies relay on the presence of wild-type Ras to be effective since oncogenic Ras transmits proliferative and antiapoptotic signals independently of the EGFR activation [22,24]. For that reason, treatments with anti-EGFR drugs such as cetuximab and panitumumab have incorporated routine assessment of *KRAS *status prior to administration [27]. The effectiveness of EGFR inhibitors in metastatic colon cancer has been reported in several studies measuring the response rate (RR) and DFS [27-35]. Importantly, the effectiveness of both drugs is limited to those tumors harboring no oncogenic mutations in *KRAS *[28].

Since most basal-like tumors express EGFR, it seemed of interest to investigate the mutational status of *KRAS *in such tumors to provide scientific evidence for the evaluation of anti-EGFR therapies in the management of triple-negative breast cancer. Our results indicate that most, if not all, triple-negative tumors harbor wild-type *KRAS*, supporting the use of EGFR inhibitors, alone or in combination with other drugs, for their treatment.

## Methods

### Samples

Tumor samples were obtained from 35 patients with early breast cancer of the basal-like subtype who had undergone treatment at the Hospital Clínico Universitario Virgen de la Victoria (HCUVV, Málaga, Spain). Tumors were classified as basal-like following the criteria stablished by Nielsen *et al*., i.e., expression of cytokeratins 5/6 and/or EGFR together with lack of expression of ER and HER2 [20]. These criteria have demonstrated 76% sensitivity and 100% specificity for the identification of basal-like breast tumors as defined by gene expression profiling. The corresponding formalin-fixed, paraffin-embedded tissues were obtained from the Pathology Department at HCUVV and processed by immunohistochemistry to check the expression of ER, EGFR and cytokeratins. Tumor areas were marked by direct visualization in 5 serial 10-μM-thick sections and manually microdissected with a razor blade. To obtain genomic DNA, dissected samples were disolved in xylene to remove paraffin and processed with the QIAamp DNA FFPE Tissue kit (Qiagen) following the manufacturer's instructions. DNA was quantitated spectrophotometrically by measuring absorbance at λ =260 nm in a Nanodrop system. All DNA samples included in this study had an A_260_/A_280 _ratio higher than 1.8. As a positive control for the mutation analysis, we also included genomic DNA prepared from two colon cancer biopsies known to be positive for *KRAS *mutation. Experimental procedures were approved by the Scientific and Ethical Review Board of the HCUVV.

### Immunohistochemistry

Immunohistochemical staining was performed on 3-μm sections of paraffin blocks containing tumour tissue. HER2 immunohistochemistry was performed following the instructions included in the HercepTest™ kit (Dako). For all other antigens, epitopes were retrieved by microwaving the sections in citrate buffer pH 6.0 for 20 min. Immunohistochemistry was carried out in a Tech Mate Horizon autoimmunostainer (Dako, Copenhagen, Denmark) using the Dako Real EnVision system for signal detection. The following antibodies were from Dako: estrogen receptor (clone 1D5), progesterone receptor (clone PgR636) and HER2 (HercepTest™). The anti-CK5/6 antibody (clone D5/16B4) was from Boehringer Biochemica). EGFR expression was determined with the EGFR pharmaDx™ kit for autostainer (Dako). For ER and PR immunoreactivity, the cut-off value of 10% was used to divide cases into negative and positive groups. HER2 expression was scored following the guidelines of the HercepTest™ kit and interpreted as negative when the staining intensity was 0 or 1+ and positive when it was 2+ or 3+. Membrane staining was used as the evaluable parameter to determine EGFR expression with the EGFR pharmaDx™ kit. Positivity for EGFR expression was defined as any membrane staining above background level in at least 1% of tumor cells. Absence of staining was reported as negative. For the basal marker CK 5/6, positivity was defined as detection of any stained invasive malignant cells.

### Mutation analysis

The Therascreen KRAS kit (Roche Diagnostics) was used to determine the mutational status of *KRAS *in the samples. Briefly, 50-100 ng of total genomic DNA was analyzed by Real Time (RT)-PCR using mutation-specific Scorpions^® ^primers. The kit allowed detection of the following mutations: Gly12Ala (GGT>GCT)522, Gly12Asp (GGT>GAT)521, Gly12Arg (GGT>CGT)518, Gly12Cys (GGT>TGT)516, Gly12Ser (GGT>AGT)517, Gly12Val (GGT>GTT)520, Gly13Asp (GGC>GAC)532. All experiments included both a positive (each mutant DNA) and a negative (no template) control reaction. Reactions were carried out in 96-well plates in an ABI 7500 Real-Time PCR system (Applied Biosystems). Threshold cycle (C_t_) was plotted against normalized reporter (R_n_) and the ΔC_t _was calculated by the formula: ΔC_t _= C_ts_-C_tc_, were C_ts _and C_tc _are the C_t _of the sample and the positive control, respectively. Values of C_tc _were in the range of 29-35. The obtained ΔC_t _values were compared with the reference values provided in the kit to classify the samples as positive or negative for each *KRAS *mutation.

## Results and discussion

**Figure 1:**
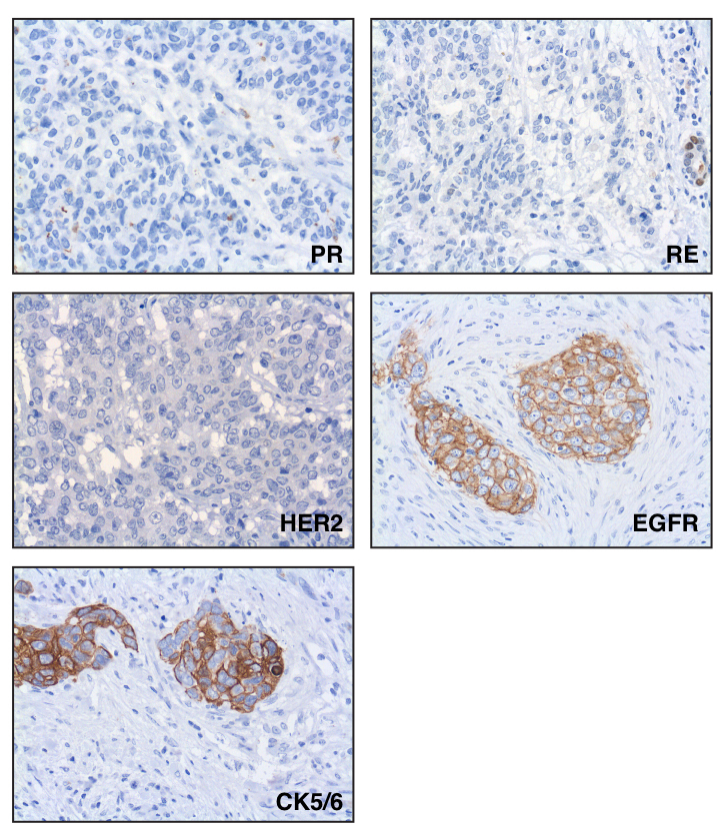
**Representative immunohistochemistry of a basal-like breast tumor showing negative staining for the hormone receptors (ER and PR) and HER2 and positive staining for EGFR and CK5/6**.

Thirty-five archived paraffin blocks containing tumor samples from different breast cancer patients were initially selected as triple-negative on the basis of their lack of immunoreactivity for the surrogate markers ER, PR and HER2 (Table 1 and Fig. 1). We then performed additional immunostaining with antibodies against CK5/6 and EGFR to identify the subgroup of basal-like tumors [20]. Such analysis revealed that 77.1% (27/35) of the tumors were basal-like. Table 2 summarizes the immunohistological characteristics of all tumors analyzed in this study. Expression of CK5/6 was detected in 63.0% of all tumors classified as basal-like while 92.6% stained positive for EGFR (Table 2). The observed proportion of basal-like tumors expressing CK5/6 is in good agreement with that reported by other groups [9,20] however, we found a higher frequency of EGFR expression when compared with the 57% and 27% values reported by Nielsen *et al*. [20] and Kreike *et al*. [9], respectively. Of note, the EGFR status in breast cancer has not been examined as extensively as in other types of cancer and its reported overexpression ranges from 14% to 91% [36-38]. EGFR expression has been associated with *BRCA1*-mutated tumors and basal-like phenotype in several studies [37,39-41]. In addition, some observations suggest that EGFR upregulation is an early event in breast tumorigenesis since EGFR overexpression can be observed in premalignant lesions [37]. As has been suggested by some investigators, a more detailed study on the activation status and subcellular localization of wild-type EGFR, in both primary and metastatic tumors, is needed to evaluate EGFR expression as a predictive marker for response to anti-EGFR therapies [42].

**Table 1:** Immunohistochemical data.

Sample #	ID	CK5/6*	EGFR*	Basal-like^=^
1	757688	-	+	yes
2	767740	+	+	yes
3	832343	-	+	yes
4	608891	+	+	yes
5	857666	+	-	yes
6	804529	+	+	yes
7	555943	+	+	yes
8	452803	-	+	yes
9	481252	+	+	yes
10	222867	-	-	no
11	402341	-	+	yes
12	760011	+	+	yes
13	834492	-	+	yes
14	778794	-	-	no
15	717674	+	+	yes
16	768943	-	-	no
17	438696	-	+	yes
18	CH	+	+	yes
19	198346	+	+	yes
20	853477	+	+	yes
21	265886	-	-	no
22	841511	+	+	yes
23	708805	-	+	yes
24	856202	-	+	yes
25	841511	+	+	yes
26	852333	+	+	yes
27	405573	+	-	yes
28	194302	-	-	no
29	108707	-	-	no
30	43742	+	+	yes
31	560504	-	-	no
32	779157	-	+	yes
33	107512	-	-	no
34	772351	-	+	yes
35	844953	+	+	yes

*positivity detected by immunohistochemistry

^=^basal-like = CK5/6+ and/or EGFR+

Abbreviations: ID, identification

**Table 2:** Frequency of immunostaining and *KRAS *mutations among breast cancer tumors.

Subtype	Samples	CK5/6+ (%)	EGFR+ (%)	CK/EGFR+ (%)*	*KRAS *mut (%)
TN	35	17 (48.6)	25 (71.4)	15 (42.8)	0 (0.0)
BS	27	17 (63.0)	25 (92.6)	15 (55.5)	0 (0.0)

*Positive staining for both CK5/6 and EGFR

Abbreviations: TN, triple-negative; BS, basal-like; CK, cytokeratin 5/6; *KRAS *mut, mutant *KRAS*

Although the subtyping of our group of 35 tumor samples did not emerged from gene expression profiling data, they were defined as triple-negative (nonbasal) or basal-like by immnostaining with a set of four surrogate markers that has been demonstrated to be 76% sensitive and 100% specific [20]. Here, we will use the term triple-negative in reference to the full set of 35 tumor samples (ER-, PR- and HER2-) while the term basal-like will be reserved for the subset of samples that, in addition to being ER-, PR- and HER2-, are positive for CK5/6 and/or EGFR staining.

Breast cancer cell lines stablished from basal-like tumors are more sensitive to EGFR inhibitors and carboplatin -alone or in combination- than those stablished from luminal tumors [32]. Both drugs have an additive effect when added in combination [32] and preclinical data argue in favor of anti-EGFR therapies in this subtype of tumors. Oncogenic Ras proteins can signal cell proliferation even in the absence of EGFR activation and thus, molecular testing of human *KRAS *mutations is of great relevance in the identification of patients that may benefit from anti-EGFR therapies. In a study reported by Hollestelle *et al*. [43]*KRAS *mutations were found in 5 out of 40 different breast cancer cell lines (13% incidence). Overall, *KRAS *mutations are infrequent in breast cancer, representing a mere 5% of all breast carcinomas [24]. However, it is not known if they are distributed randomly in all five molecular subtypes of breast cancer (luminal A, luminal B, HER2+, normal-like and basal-like) or concentrated in one or a few subtypes. In particular, ~60% of the basal-like tumors express EGFR and thus, they are an attractive target for EGFR inhibitors. Thus, we wanted to investigate if molecular testing of *KRAS *mutations would serve as a prognostic factor in adjuvant therapy recommendations for basal-like breast cancer patients.

**Figure 2:**
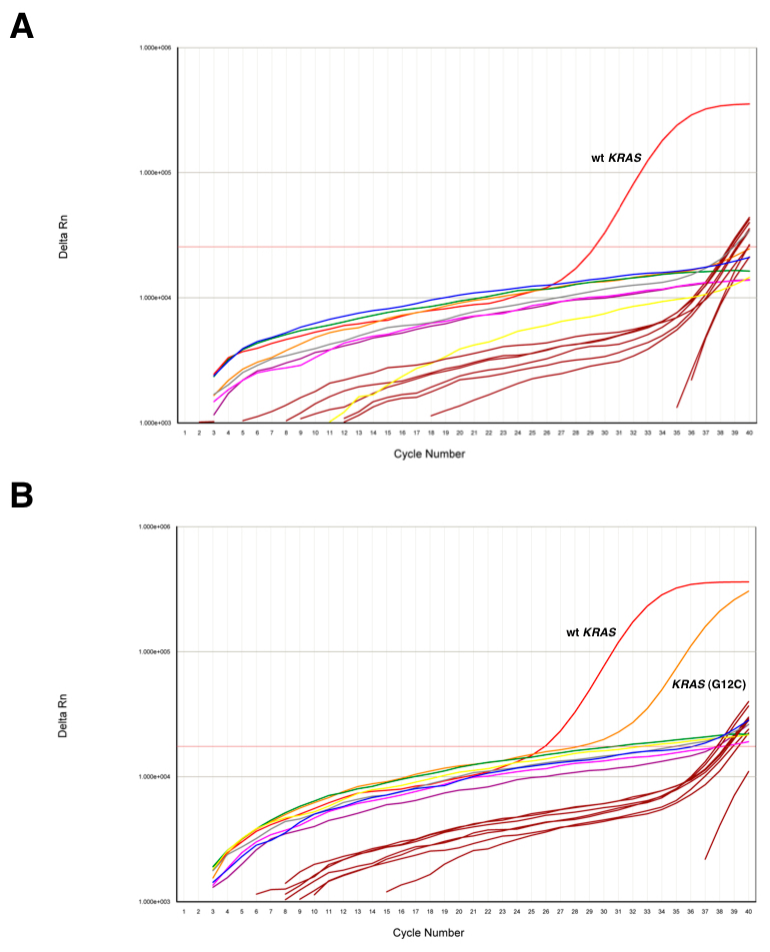
**Detection of *KRAS *mutations by RT-PCR**. *A*, The graph shows a representative amplification curve (ΔRn vs cycle) from 100 ng of genomic DNA prepared from a triple-negative tumor sample. RT-PCR reactions were performed with primers specifically designed to amplify wild-type KRAS (red) or the following mutants: Gly12Ala (green), Gly12Asp (blue), Gly12Arg (yellow), Gly12Cys (Pink), Gly12Ser (brown), Gly12Val (purple), Gly13Asp (grey). Brown lines correspond to the amplification profile of an internal control included in each reaction to check for false positives. *B*, As a positive control, genomic DNA was obtained from a colon carcinoma biopsy and subjected to RT-PCR as in A. Note the presence of the Gly12Cys mutation.

To that aim, total genomic DNA obtained from each paraffin-embedded tumor was subjected to RT-PCR reactions with primers specifically designed to amplify and detect seven cancer-related somatic mutations in codons 12 and 13 of human *KRAS *[44,45]. Notably, none of the DNA samples could function as template for amplification of the *KRAS *oncogenic mutations, indicating that the full set of 35 triple-negative tumors expressed the wild-type protein (Table 2 and Fig. 2). As such, the wild-type *KRAS *gene could be amplified and detected in all 35 DNA samples. Also, genomic DNA from a colon carcinoma known to harbor a Gly12Cys mutation in *KRAS *could be amplified and the mutation detected by RT-PCR (Fig. 2). This result indicates that the lack of *KRAS *mutations observed in the breast tumor samples were not due to a deficiency in the assay (Fig. 2). Therefore, we found no evidence of *KRAS *somatic mutations in human triple-negative tumors as measured by a standarized assay [44,45]. We cannot exclude the possibility that a minimal number of cells, below the detection limit of the assay (< 1% of tumor cells) harbor mutations in *KRAS*, however, we used the same diagnostic assay currently included in the clinical practice to select colorectal patients for anti-EGFR treatments. It is well known that *KRAS *mutations are infrequent in breast cancer [24] and our data further indicates that they are not distributed homogeneously and are uncommon, if not absent, in triple-negative tumors. In a recent study aimed at the identification of EGFR-associated expression profiles in different breast cancer subtypes, Perou and co-workers mentioned that, as a control, they sequenced 96 breast tumors and found no common mutations in *BRAF*, *HRAS *and *KRAS *[32]. While our results are in agreement with such findings, they represent, to the best of our knowledge, the first attempt to directly determine the incidence of *KRAS *mutations in basal-like breast tumors and to discuss them in the context of anti-EGFR therapies.

Two randomized phase II trials have evaluated the role of cetuximab in triple negative breast cancer. In the TBCRC 001 study, eligible, pretreated patients received the anti-EGFR monoclonal antibody cetuximab alone, with planned crossover to cetuximab plus carboplatin upon progression (arm 1) or cetuximab combined with carboplatin from the very begining (arm 2). Monotherapy with cetuximab showed low clinical benefit (CB) and RR (10% and 6%, respectively) and was cancelled early due to lack of efficacy. Moreover, the combination of cetuximab plus carboplatin achieved a modest activity: 17% RR, and 31% CB [46]. A different phase II trial showed a higher RR in triple-negative patients treated with the combination of irinotecan plus carboplatin and cetuximab versus those treated with irinotecan plus carboplatin (49% vs 30%) [46]. Both trials included unselected patients with heavily pretreated tumors. It should be noted that although most basal-like cancers do not express ER and HER2, 15% to 45% are reported to express at least one of these markers. On the other hand, not all triple negative cancers are of basal-like profile, only approximately 85% of ER- and Her2- cancers are classified as basal-like by microarray analysis [20].

Besides *KRAS*, alterations in the phosphatidylinositol 3-kinase (PI3K) pathway have been described in several types of cancer [47,48]. In particular, activating mutations in *PIK3CA*, the gene encoding for the p110α catalytic subunit of PI3K, confers resistance to cetuximab-induced cell cycle arrest in colon cancer cell lines [49]. The cells are maximally resistant when *KRAS *and *PIK3CA *are mutated simultaneously [49]. The *PIK3CA *mutation frequency in breast cancer reportedly varies between 8% and 40% [50-53]. Kalinsky *et al*. found *PI3KCA *mutations in 32.5% of invasive breast primary tumors in a large cohort of 590 samples [52] and, interestingly they correlated with older age at diagnosis, lower tumor grade and stage, and lymph node negativity. In addition, patients with *PIK3CA *mutations had improved OS and breast cancer-specific survival [52]. In a different study with a smaller cohort of 292 breast cancer patients, activation of the PI3K pathway (by genetic alterations in the *PIK3CA*, *PTEN *or *AKT *genes) was found to be significantly associated with a basal-like phenotype, high tumor grade and death from breast cancer. However, *PIK3CA *mutations alone did not correlate with any clinicopathological parameter [54]. Other studies have also reported contradictory -both favorable and poor- patient outcomes associated with *PIK3CA *mutations in breast cancer [51,55]. While we have not addressed the mutational status of the PI3K pathway, the results from Kalinsky *et al*. suggest that activating mutations in *PIK3CA *will not confer resistance to anti-EGFR therapies. In fact, mutant cancer cells could be more sensitive to these type of agents. Alternatively, identifying PIK3CA activating mutations in older patients could benefit them by minimizing the therapy. Additional studies are needed to clarify this issues.

In summary, despite the fact that most basal-like tumors included in our study expressed EGFR, we found no evidence of oncogenic mutations in *KRAS*. Therefore, we conclude that testing for *KRAS *mutations is not necessary as a diagnostic factor in the treatment of basal-like breast cancer. Furthermore, the wild-type status of *KRAS *observed in all samples analyzed here indicate that anti-EGFR therapeutic strategies, such as those using monoclonal antibodies (cetuximab, panitumumab) or small molecule inhibitors (gefitinib, erlotinib), may be of potential benefit in the treatment of basal-like breast cancer.

## Conclusions

Since we found no incidence of oncogenic *KRAS *mutations in basal-like tumors, our results indicates that therapies based on EGFR inhibition may be of benefit in the treatment of this particularly agressive subtype of breast tumors.

## List of abbreviations

RR: response rate; DFS: disease-free survival; OS: overall survival; RT-PCR: real-time polymerase chain reaction; ER: estrogen receptor; PR: progesterone receptor; EGFR: epidermal growth factor receptor; HER2: human epidermal growth factor receptor 2; CK: cytokeratin.

## Competing interests

The authors declare that they have no competing interests.

## Authors' contributions

ASM contributed to the study's design, analysis of data and manuscript drafting. EG, VL and LPR processed the tumor samples. LV and NR participated in the study's design. JL contributed to the study's design, performed the mutational analysis and wrote the manuscript. EA contributed to the study's conception and design, interpretation of the data and manuscript writing. All authors read and approved the final manuscript.

## Pre-publication history

The pre-publication history for this paper can be accessed here:

http://www.biomedcentral.com/1471-2407/10/136/prepub
